# Thyroid autoimmunity and vitamin D: Effects on *in vitro* fertilization/intracytoplasmic sperm injection laboratory outcomes

**DOI:** 10.3389/fendo.2022.1079643

**Published:** 2022-12-15

**Authors:** Yalong Liu, Zining He, Ning Huang, Lin Zeng, Fangyin Meng, Rong Li, Hongbin Chi

**Affiliations:** ^1^Center for Reproductive Medicine, Department of Obstetrics and Gynecology, Peking University Third Hospital, Beijing, China; ^2^National Clinical Research Center for Obstetrics and Gynecology, Peking University Third Hospital, Beijing, China; ^3^Key Laboratory of Assisted Reproduction (Peking University), Ministry of Education, Beijing, China; ^4^Beijing Key Laboratory of Reproductive Endocrinology and Assisted Reproductive Technology, Peking University Third Hospital, Beijing, China; ^5^Clinical Epidemiology Research Center, Peking University Third Hospital, Beijing, China; ^6^California Dream Fertility Center, Irvine, CA, United States

**Keywords:** thyroid immunity, follicular fluid, vitamin D, anti-Mullerian hormone, ovarian reserve

## Abstract

This prospective cohort study aimed to determine the effects of thyroid autoimmunity, serum/follicular fluid vitamin D levels, and vitamin D receptor expression in granulosa cells on laboratory outcomes of *in vitro* fertilization/intracytoplasmic sperm injection. The study included 206 women with or without thyroid autoimmunity undergoing *in vitro* fertilization/intracytoplasmic sperm injection ovarian stimulation cycles. The primary outcomes in thyroid autoimmunity and non-thyroid autoimmunity patients with high or low follicular fluid vitamin D levels (high vitamin D level, ≥20 ng/mL; low vitamin D level, <20 ng/mL) were the number of oocytes retrieved and quality of embryos. The secondary outcomes were the association between serum and follicular fluid vitamin D levels and vitamin D receptor expression in granulosa cells. Our study revealed that thyroid autoimmunity was associated with fewer good-quality embryos but not oocytes (*p =* 0.010). The vitamin D level in the follicular fluid was significantly correlated with that in the serum (*p* < 0.001, *r* > 0.5). The study populations in the thyroid autoimmunity and non-thyroid autoimmunity groups were divided into two subgroups based on high/low serum/follicular fluid vitamin D levels. There was no significant difference in the number of retrieved oocytes and good-quality embryos between the subgroups with high or low vitamin D levels (*p* > 0.05), and the incidence of thyroid autoimmunity was comparable between the subgroups (*p* > 0.05). Linear regression analysis indicated that thyroid autoimmunity had a negative effect on the number of healthy embryos (*p =* 0.038). Reverse transcription-polymerase chain reaction results indicated that vitamin D receptor expression in granulosa cells was positively correlated with follicular vitamin D levels in the thyroid autoimmunity (*p* = 0.0002) and non-thyroid autoimmunity (*p* < 0.0001) groups. The current findings suggest that thyroid autoimmunity may have a more detrimental effect on *in vitro* fertilization/intracytoplasmic sperm injection laboratory outcomes than vitamin D.

## 1 Introduction

Vitamin D, a prohormone classified as a vitamin for the first time in the 20th century, is implicated in numerous biological processes (1). Vitamin D, also known as calcitriol or 1,25(OH)2 vitamin D, regulates calcium and phosphate homeostasis, cell proliferation, and differentiation. Vitamin D modulates the immunological, neurological, circulatory, and female reproductive systems (2). Granulosa cells (GCs), which are surrounded by the follicle, express the vitamin D receptor (VDR) (3, 4) and may provide insight into alterations in the follicular microenvironment caused by fluctuations in follicular fluid (FF) vitamin D levels. FF vitamin D has been linked to follicle development (5), oocyte and embryo maturation (6), and the success of *in vitro* fertilization/intracytoplasmic sperm injection (IVF/ICSI) (7).

Thyroid autoimmunity (TAI) is characterized by reduced 25(OH)D levels (8, 9). TAI is the predominant cause of primary hypothyroidism resulting from the targeting of thyroid peroxidase (TPO) or thyroglobulin (TG) by autoantibodies, which may ultimately lead to thyroid tissue destruction (10). Supplementation significantly reduces autoantibody titers in patients with vitamin D deficiency (11). TAI may also affect the female reproductive system. Miscarriage rates have been found to be higher in the first trimester of pregnancy among euthyroid women with thyroid antibody (TPO- and TG-positive) (12). Further, TAI is associated with various gynecological issues, including unexplained infertility (13, 14) and IVF failure (14, 15). Despite its importance, there are few studies focusing on the molecular mechanism by which TAI affects oocyte development and maturation.

In light of this understanding, we conducted a prospective cohort study to obtain deeper insights into the effects of TAI on serum (S) and FF vitamin D levels in infertile patients with or without TAI who were undergoing IVF/ICSI treatment.

## 2 Materials and methods

### 2.1 Study design and setting

This prospective single-center cohort study was conducted at the Reproductive Center of Peking University Third Hospital from April 2021 to December 2021. The Peking University Third Hospital Medical Science Research Ethics committee approved the study protocol (registration no. M2021189). The patients provided written informed consent.

### 2.2 Participants

Female patients undergoing IVF/ICSI cycles were invited to participate in this study. The following patients were included in the study: 1) women between the ages of 20 and 40 years; 2) fresh embryo transfer (ET) recipients; and 3) those with male or tubal factor infertility. Patients with any of the following conditions were excluded: 1) any reproductive endocrinological disorders leading to infertility, including polycystic ovary syndrome, endometriosis, premature ovarian failure, hyperprolactinemia, and diminished ovarian reserve; 2) previous thyroidectomy; 3) cases complicated by autoimmune disorders; 4) cardiopulmonary, liver, and kidney diseases; 5) vitamin D supplementation; 6) *in vitro* fertilization failure over three times; 7) pelvic/intrauterine adhesion or untreated hydrosalpinx; 8) recurrent abortion; 9) uterine malformation; or 10) uterine myoma (multiple, submucous, or intramural myoma > 4 cm). A total of 103 patients with TAI were recruited for the study, and another 103 non-TAI patients were randomly selected to match the sample size of the TAI group.

All patients underwent a standardized and controlled ovarian stimulation regimen, oocyte retrieval, and fertilization, followed by fresh ET IVF/ICSI cycles. The protocols were as follows:

1) Antagonist protocol: Recombinant gonadotropins were initiated on the second day of the menstrual cycle, and gonadotropin-releasing hormone (GnRH) antagonist was administered daily when at least one follicle reached 12 mm in diameter. The treatment was repeated until human chorionic gonadotropin (HCG) was administered. 2) Short-term protocol: Short-acting GnRH agonist and recombinant gonadotropins were injected for ovarian stimulation. 3) Long-term protocol: Recombinant gonadotropins were injected for ovarian stimulation after downregulation was achieved by mid-luteal administration of long-acting GnRH agonist. 4) Ultralong-term protocol: Recombinant gonadotropins were injected for ovarian stimulation starting between day 28 and day 30 of the menstrual cycle after downregulation was achieved using long-acting GnRH agonist on the first day of that cycle.

The individualized dose of gonadotropins was based on patient age, BMI, and anti-Mullerian hormone levels. Recombinant HCG (250 μg; Eiser, Serono, Germany) was administered to trigger oocyte maturation when at least two follicles reached 18 mm in diameter. Oocyte retrieval was performed 34–36 h after HCG administration. Insemination was performed at 4–6 h after oocyte retrieval using a routine IVF method or ICSI injection, according to the sperm quality. One to two embryos or blastocysts were transferred 3 or 5 days after oocyte retrieval.

### 2.3 Serum collection

A blood sample was collected on the second day of the menstrual cycle from each participant to evaluate the total vitamin D concentration.

### 2.4 Follicular fluid collection

Before measuring vitamin D levels, the FF supernatant was retrieved from the largest follicle, centrifuged, and frozen at -80°C. An FF sample collected from the follicular aspirates of each participant was used to evaluate total vitamin D concentrations and to isolate GCs for *VDR* expression analysis using real-time polymerase chain reaction (RT-PCR).

### 2.5 RT-PCR

Total RNA was extracted from GCs isolated from the FF using TRIzol reagent (Life Technologies, Beijing, China), according to the manufacturer’s instructions. A cDNA synthesis kit (Thermo Fisher Scientific, Beijing, China) was employed for RNA reverse transcription. *VDR* expression was detected using SYBR green (QuantiTect SYBRVR Green PCR Kits, QIAGEN, Beijing, China) and an Applied Biosystems (Waltham, MA, USA) Quant Studio3 Real-Time PCR System with 96-well optical reaction plates. The following primers were used: β-actin F, TGCCCATCTACGAGGGGTAT; β-actin R, CTTAATGTCACGCACGATTTCC; *VDR* F, GGTGGAGGGAGCCATCCTT; *VDR* R, TGGGACAGCTCTAGGGTCACA.

### 2.6 Study end points

The primary outcomes were the number of oocytes retrieved and good-quality embryos in the TAI/non-TAI group with high/low FF vitamin D level (high vitamin D [HVD] level, ≥20 ng/mL; low vitamin D [LVD] level, <20 ng/mL). The secondary outcomes were the associations between serum and FF vitamin D levels and between FF vitamin D levels and *VDR* expression in GCs. The HVD level was set at ≥20 ng/mL based on a research study by Chao and colleagues (9). In their study, vitamin D levels were analyzed in 5,230 Chinese participants and deficiency (defined as <20 ng/mL) was reported in over 70% of the participants with or without Hashimoto’s thyroiditis. Thus, we chose to define HVD as a level ≥20 ng/mL.

### 2.7 Laboratory testing

Blood samples for thyroid hormone (TH) testing were collected within six months prior to the initiation of controlled ovarian stimulation. Serum and FF total 25(OH)D concentrations were measured on the second day of the menstrual cycle and the day of ovum retrieval, respectively. Serum thyroid-stimulating hormone (TSH), free thyroxin (FT4), TPO antibody (TPOAb), and TG antibody (TGAb) levels were measured using a fully automated ADVIA Centaur XP chemiluminescence immunoassay analyzer (Siemens Healthcare Diagnostics). Serum/FF vitamin D levels were determined using a fully automated Elecsys Vitamin D total chemiluminescence immunoassay analyzer (Roche Diagnostics GmbH, Pleasanton, CA, USA). The reference ranges for TSH and FT4 were 0.55–4.78 μIU/mL and 0.89–1.80 ng/dL, respectively. The vitamin D measuring range was 3–100 ng/mL (defined by the limit of detection and the master curve maximum value). Values below the limit of detection were reported as <3.0 ng/mL. Values above the measuring range were reported as >100 ng/mL or up to 200 ng/mL for 2-fold diluted samples. Embryos were evaluated on the third or fifth day after fertilization. Good-quality embryos were all developed from two pronuclei zygotes and met the following criteria: 1) they had more than five blastomeres, 2) size difference was less than 20%, and 3) fragmentation was less than 50%. Good-quality blastocysts met the criteria generally used in our center (16).

### 2.8 Categorization

Patients were divided into TAI and non-TAI groups based on TPOAb or TGAb positivity (TPOAb or TGAb levels <60 IU/mL were considered negative), and then patients in each group were further allocated into two subgroups according to their S/FF vitamin D status (≥20 ng/mL and <20 ng/mL).

### 2.9 Statistical analysis

Mean (standard deviation [SD]), and median (interquartile range) were used to describe normally and non-normally distributed continuous data, respectively. *VDR* expression data were presented as the mean (standard error of the mean [SEM]). Categorical data are shown as the number of cases (percentage). Student’s t-test or one-way analysis of variance and the chi-square test were used to compare continuous and categorical variables, respectively. Continuous variables without normal distributions were compared using the Mann–Whitney U test. The correlations between serum and FF 25(OH)D levels were determined using Spearman rank correlation analysis. Linear regression was performed to analyze the association between the number of retrieved oocytes, the number of good-quality embryos, and other relevant factors. A two-sided *p*-value <0.05 was considered statistically significant. Analyses were performed using SPSS version 24.0.

## 3 Results

Demographics of the study population based on TAI and non-TAI are presented in **Table 1**. Patients in the TAI group were younger than those in the non-TAI group (*p* = 0.009), and the TAI group had fewer good-quality embryos (*p* = 0.010). The prevalence of primary infertility was 70% in the TAI group and 56% in the non-TAI group, with significant differences (*p* = 0.045).

**Table 1:** Baseline characteristics and *in vitro* fertilization data of patients based on TAI and non-TAI.

Characteristic	TAI (n = 103)	Non-TAI (n = 103)	*p*-value
Age, median (IQR)	32 (30–34)	34 (31–36)	0.009
Body mass index, median (IQR), kg/m^2^	22.3 (20.4–24.0)	21.6 (20.0–24.0)	0.345
Type of infertility, No. (%)			0.045
Primary	70 (68.0)	56 (54.4)	
Secondary	33 (32.0)	47 (45.6)	
Basal FSH, median (IQR), mIU/mL^a^	6.69 (5.14–7.75)	6.42 (5.20–7.73)	0.454
AMH, median (IQR), ng/mL	2.60 (1.66–3.85)	2.42 (1.54–3.74)	0.385
FT4, median (IQR), ng/dL	1.24 (1.14–1.35)	1.25 (1.12–1.34)	0.678
TT4, median (IQR), μg/dL	8.00 (6.70–9.20)	7.60 (6.90–8.60)	0.374
TSH, median (IQR), mIU/L	1.99 (1.46–3.13)	2.06 (1.47–2.70)	0.746
Serum 25(OH) D level, median (IQR), ng/mL	20.1 (14.1–26.5)	20.3 (15.1–25.8)	0.796
Serum 25(OH) D status, No. (%)			1.000
≥20 ng/mL	52 (50.5)	52 (50.5)	
<20 ng/mL	51 (49.5)	51 (49.5)	
Follicular fluid 25(OH) D level, median (IQR), ng/mL	21.6 (15.3–28.6)	20.8 (16.5–30.0)	0.646
Antral follicle count in both ovaries, median (IQR)	11.0 (8.0–15.0)	10.0 (8.0–15.0)	0.519
No. of oocytes retrieved per cycle, median (IQR)	10.0 (6.0–15.0)	13.0 (6.0–18.0)	0.110
Fertilization rate, median (IQR), %	69.2 (50.0–83.3)	72.7 (56.3–87.8)	0.345
No. of good-quality embryos per cycle, median (IQR)^b^	3.0 (1.0–6.0)	4.0 (2.0–7.0)	0.010

FSH, follicle-stimulating hormone; AMH, anti-Mullerian hormone; FT4, free thyroxine; TT4, total thyroxine; IQR, interquartile range; TAI, thyroid autoimmunity; TSH, thyroid-stimulating hormone.

a Basal FSH was measured on the second day of the menstrual cycle.

b Embryos were evaluated on the third or fifth day after fertilization. Good-quality embryos were all developed from two pronuclei zygotes and met the following criteria: 1) had more than five blastomeres, 2) size difference was less than 20%, and 3) fragmentation was less than 50%. Good-quality blastocysts met the criteria generally used in our center (16).

**Figure 1:**
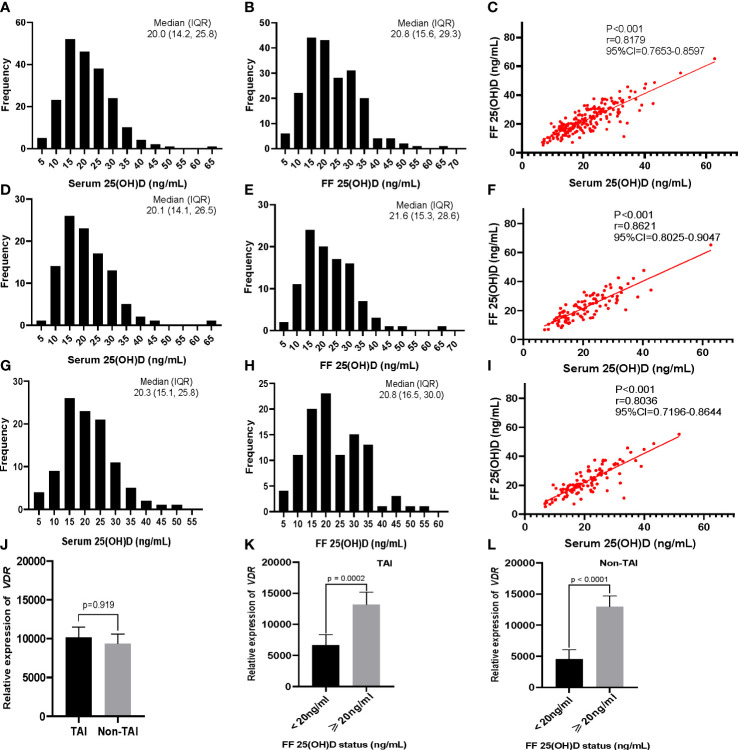
Distribution and correlation of serum and follicular fluid (FF) 25(OH)D concentrations in participants, and vitamin D receptor (*VDR*) gene expression in patient granulosa cells determined using RT-PCR. Distribution of **(A)** serum and **(B)** FF levels of 25(OH) D in all 206 participants. **(C)** Correlation of serum and FF levels of 25(OH)D. **(D–F)** Corresponding distribution and correlation results for the TAI (n = 103) and **(G–I)** non-TAI groups (n = 103). **(J)** *VDR* expression in the TAI and non-TAI groups. **(K, L)** Level of *VDR* expression based on the FF 25(OH) D status (<20 ng/mL and ≥20 ng/mL) for **(K)** TAI and **(L)** non-TAI patients. The correlations between serum and FF 25(OH)D levels were determined using Spearman rank correlation analysis. The Mann–Whitney U test was used to compare the relative expression of *VDR* in different groups. Data are presented as the mean ± SEM.

No significant differences in serum and FF 25(OH)D levels were observed between the TAI and non-TAI groups. The distribution of total 25(OH)D levels is depicted in **Figure 1**; FF 25(OH)D levels were strongly correlated with serum 25(OH)D levels (*p* < 0.001, *r* > 0.5). In addition, the study population was divided into two subgroups based on S/FF vitamin D status (HVD, ≥20 ng/mL and LVD, <20 ng/mL) and analyzed as HVD-S, HVD-FF, LVD-S, and LVD-FF (**Tables 2**, **3**). The incidence of TAI was comparable between the HVD-S/FF and LVD-S/FF groups. Patients in the HVD-S group presented with an increased incidence of poor ovarian reserve (POR, anti-Mullerian hormone (AMH) < 1.2 ng/mL) (17) (*p* = 0.007), but there was no significant difference in AMH levels between the HVD-S and LVD-S groups (*p* = 0.134) (**Table 2**). Patients in the HVD-FF group tended to have significantly lower AMH levels than those in the LVD-FF group (*p* = 0.032), with POR prevalence rates of 19.3% and 8.7% in the HVD-FF and LVD-FF groups, respectively (*p* = 0.032) (**Table 3**). The number of retrieved oocytes and healthy embryos did not differ significantly between the HVD and LVD groups (**Tables 2**, **3**). Linear regression analysis indicated that TAI had a negative effect on achieving an increased number of good-quality embryos (*p =* 0.038) (**Table 4**).

**Table 2:** Baseline characteristics and *in vitro* fertilization data of patients based on serum vitamin D status.

Characteristics	≥20 ng/mL) (n = 104)	<20 ng/mL (n = 102)	*p*-value
Age, median (IQR)	33 (30–37)	33 (30–35)	0.717
Body mass index, median (IQR), kg/m^2^	22.0 (20.4–24.3)	21.8 (19.8–23.8)	0.192
TAI, No. (%)	52 (50)	51 (50)	1.000
Type of infertility, No. (%)			0.691
Primary	65 (62.5)	61 (59.8)	
Secondary	39 (37.5)	41 (40.2)	
Basal FSH, median (IQR), mIU/mL^a^	6.52 (5.11–8.13)	6.55 (5.25–7.42)	0.472
AMH, median (IQR), ng/mL	2.32 (1.41–3.66)	2.66 (1.69–3.92)	0.134
AMH <1.2 ng/mL No. (%)	22 (21.2)	8 (7.8)	0.007
FT4, median (IQR), ng/dL	1.23 (1.14–1.35)	1.25 (1.12–1.35)	0.882
TT4, median (IQR), μg/dL	7.70 (6.75–8.90)	7.90 (6.95–8.95)	0.519
TSH, median (IQR), mIU/L	1.96 (1.40–2.72)	2.08 (1.52–2.94)	0.339
Follicular fluid 25(OH)D level, median (IQR), ng/mL	29.0 (23.2–34.2)	16.4 (12.2–19.7)	<0.001
Antral follicle count in both ovaries, median (IQR)	10.0 (8.0–15.5)	11.0 (8.0–15.0)	0.631
No. of oocytes retrieved per cycle, median (IQR)	10.5 (6.0–16.0)	12.0 (6.5–16.0)	0.618
Fertilization methods, No. (%)			0.523
IVF	48 (46.2)	54 (52.9)	
ICSI	52 (50.0)	43 (42.2)	
IVF+ICSI	4 (3.8)	5 (4.9)	
Fertilization rate, median (IQR), %	70.0 (50.0–81.8)	73.3 (58.6–88.4)	0.162
No. of good-quality embryos per cycle, median (IQR)^b^	3.0 (1.0–7.0)	4.0 (2.0–7.0)	0.771

FSH, follicle-stimulating hormone; AMH, anti-Mullerian hormone; FT4, free thyroxine; TT4, total thyroxine; IQR, interquartile range; TAI, thyroid autoimmunity; TSH, thyroid-stimulating hormone. IQR, interquartile range.

a Testing for basal FSH was measured on the second day of the menstrual cycle.

b Embryos were evaluated on the third or fifth day after fertilization. Good-quality embryos were all developed from two pronuclei zygotes and met the following criteria: 1) they had more than five blastomeres, 2) size difference was less than 20%, and 3) fragmentation was less than 50%. Good-quality blastocysts met the criteria generally used in our center (16).

**Table 3:** Baseline characteristics and *in vitro* fertilization data of patients based on follicular fluid vitamin D status.

Characteristic	≥20 ng/mL (n = 114)	<20 ng/mL (n = 92)	*p*-value
Age, median (IQR)	33 (30–37)	32 (30–34)	0.193
Body mass index, median (IQR), kg/m^2^	22.0 (20.2–24.4)	21.8 (19.9–23.7)	0.378
TAI, No. (%)	55 (48.2)	48 (52.2)	0.575
Type of infertility, No. (%)			
Primary	67 (58.8)	59 (64.1)	0.433
Secondary	47 (41.2)	33 (35.9)	
Basal FSH, median (IQR), mIU/mL^a^	6.59 (5.14–7.92)	6.49 (5.20–7.41)	0.414
AMH, median (IQR), ng/mL	2.26 (1.56–3.35)	2.78 (1.68–4.10)	0.032
AMH <1.2 ng/mL No. (%)	22 (19.3)	8 (8.7)	0.032
FT4, median (IQR), ng/dL	1.20 (1.12–1.32)	1.26 (1.14–1.36)	0.077
TT4, median (IQR), μg/dL	7.70 (6.80–8.80)	7.90 (7.0–9.10)	0.434
TSH, median (IQR), mIU/L	1.98 (1.29–2.77)	2.05 (1.65–2.98)	0.180
Antral follicle count in both ovaries, median (IQR)	10.0 (8.0–14.0)	12.0 (9.0–15.0)	0.088
No. of oocytes retrieved per cycle, median (IQR)	10.5 (6.0–16.0)	12.0 (7.0–16.0)	0.292
Fertilization methods, No. (%)			0.615
IVF	53 (46.5)	49 (53.3)	
ICSI	56 (49.1)	39 (42.4)	
IVF+ICSI	5 (4.4)	4 (4.3)	
Fertilization rate, median (IQR), %	70.0 (50.0–85.2)	72.8 (58.6–87.3)	0.337
No. of good-quality embryos per cycle, median (IQR)^b^	3.0 (1.0–7.0)	4.0 (2.0–7.0)	0.438

FSH, follicle-stimulating hormone; AMH, anti-Mullerian hormone; FT4, free thyroxine; TT4, total thyroxine; IQR, interquartile range; TAI, thyroid autoimmunity; TSH, thyroid-stimulating hormone.

a Basal FSH was measured on the second day of the menstrual cycle.

b Embryos were evaluated on the third or fifth day after fertilization. Good-quality embryos were all developed from two pronuclei zygotes and met the following criteria: (1) they had more than five blastomeres, (2) size difference was less than 20%, and (3) fragmentation was less than 50%. Good-quality blastocysts met the criteria generally used in our center (16).

**Table 4:** Linear regression analysis of the number of oocytes retrieved and the number of good-quality embryos.

	No. of oocytes retrieved		No. of good-quality embryos	
	Regression coefficient	*p*-value	Regression coefficient	*p*-value
Serum 25(OH)D concentration, ng/mL	-0.044	0.577	0.002	0.955
Serum 25(OH)D status (<20 ng/mL *vs*. ≥20 ng/mL)	-0.490	0.710	-0.363	0.597
Follicular fluid 25(OH)D concentration, ng/mL	-0.076	0.272	-0.015	0.669
Follicular fluid 25(OH)D status (<20 ng/mL *vs*. ≥20 ng/mL)	-0.215	0.872	-0.087	0.901
TAI *vs*. non-TAI	-1.550	0.245	-1.448	0.038
*VDR* expression	-8.659×10^-6^	0.868	4.683×10^-5^	0.082

Linear regression model also incorporated age, BMI (body mass index), FSH (follicle-stimulating hormone), LH (luteinizing hormone), E2 (estradiol), AMH (anti-Mullerian hormone), FT4 (free thyroxine), TSH (thyroid-stimulating hormone), and AFC (antral follicle count) as relevant factors.

RT-PCR results indicated that the relative expression of *VDR* in GCs was positively correlated with FF vitamin D levels in both the TAI (*p* = 0.0002) and non-TAI (*p* < 0.0001) groups. Overall, there was no significant difference in GC *VDR* expression between the TAI and non-TAI groups (**Figure 1**).

## 4 Discussion

This is the first report evaluating the relationship between TAI, S and FF vitamin D levels, and IVF laboratory outcomes in patients. In a previous study, patients with Hashimoto’s thyroiditis, which is characterized by TAI, were reported to have reduced 25(OH)D levels compared with patients without the disease. However, the participants in this earlier investigation did not include any infertile females; the participants were male and female patients aged 48.95 ± 9.06 years undergoing a health examination (9). In the present investigation, patients with a high FF vitamin D level (≥20 ng/mL) had reduced AMH levels, and the FF vitamin D level was found to be correlated with the serum vitamin D level. Linear regression analysis revealed that TAI negatively affected the number of good-quality embryos produced by patients. *VDR* expression in GCs was positively correlated with FF vitamin D levels in the TAI and non-TAI groups. These findings suggest that TAI may have a stronger negative influence on IVF/ICSI laboratory outcomes than vitamin D. The level of FF vitamin D was previously reported to be inversely associated with oocyte maturation and fertilization ability before implantation. Furthermore, high-quality embryos were more likely to mature in the FF with a low 25(OH)D concentration, resulting in higher pregnancy and delivery rates (6). Similarly, Anifandis and colleagues (18) found that the combined effects of elevated vitamin D levels and low glucose levels in the FF may have detrimental effects on IVF outcomes. Higher FF vitamin D levels have been suggested to result in lower fertilization rates (19). Although we could not confirm these findings, our study revealed that patients in the HVD group (≥20 ng/mL) had lower AMH levels and a higher incidence of POR (AMH < 1.2 ng/mL). AMH is secreted by GCs and regulates early follicular development, serving as a marker of the ovarian reserve (20). Wojtusik et al. (21) examined the effect of vitamin D on GC proliferation as well as *AMH*, *VDR*, and FSH receptor (*FSHR*) gene expression in hens. They found that *AMH* expression in GCs in small follicles was decreased by vitamin D treatment, which, on the contrary, increased GC *FSHR* expression and the proliferation of GCs. In addition, larger follicles were associated with higher *VDR* expression. Bednarska-Czerwinska and colleagues (22) described AMH levels as being negatively correlated with FF vitamin D levels. Further, Merhi et al. (23) reported a negative correlation between FF vitamin D and *AMH* and AMH receptor (*AMHR*) gene expression in GCs of reproductive-aged females. However, vitamin D has been shown to have beneficial effects in this context. Farzadi and colleagues (24) evaluated 80 patients undergoing IVF cycles and found that those with higher FF vitamin D levels tended to have a better fertilization rate. Ozkan et al. (25) reported that patients achieving clinical pregnancy had higher FF vitamin D levels, which was confirmed as an independent predictor of IVF treatment success using multivariable logistic regression analysis. A cross-sectional study of 388 premenopausal women revealed a positive correlation between the AMH level and blood vitamin D levels in women over the age of 40 years. Based on these findings, we hypothesize that vitamin D promotes favorable IVF laboratory outcomes by acting through the VDR, enhancing GC proliferation, inhibiting *AMH* expression, and upregulating *FSHR* expression, promoting oocyte recruitment and maturation.

TAI is a typical T-cell-mediated autoimmune disease characterized by the presence of TPOAb and/or TGAb in serum (12). The present study revealed that patients with TAI were generally younger, had a higher incidence of primary infertility, and showed a reduced number of good-quality embryos. These findings support the association between unexplained infertility and TAI. Our team previously conducted a large-scale retrospective cohort study involving 1,556 patients with infertility who received their first IVF/ICSI treatment and achieved fresh ET at the Reproductive Center of Peking University Third Hospital. TAI was associated with a lower number of retrieved oocytes (26). A cross-sectional study of 122 patients, aged 20–40 years, who received IVF/ICSI treatment, focused on the follicular microenvironment of TAI patients. Results revealed that the levels of three chemokines (CXCL9/10/11) and one cytokine (IFNγ) as well as the percentage of CXCR3+ T lymphocytes were elevated, suggesting the occurrence of immunological imbalance (27). Women who experienced multiple miscarriages were reported to have increased numbers of CD5/20+ B cells. Moreover, abnormal T lymphocyte function, including an increase in endometrial T cell numbers, has been observed in women with TAI (28). TAI is characterized by intrathyroidal infiltration of B and T lymphocytes with CD4+ type 1 T helper (Th1) subtype predominance (29). As stated above, vitamin D significantly modulates the immune system. VDR can be detected in most immune cells, including T cells, B cells, and antigen-presenting cells (APCs), such as dendritic cells and macrophages. Vitamin D may impede dendritic cell differentiation and maturation by inhibiting major histocompatibility complex class II expression on APCs, resulting in decreased antigen presentation and lower T cell activation. Moreover, vitamin D may suppress adaptive immunity and promote tolerance by inhibiting Th1 cell proliferation and promoting Th2 cytokine production. Further research on larger cohorts is necessary to elucidate the mechanism by which vitamin D and TAI influence IVF/ICSI laboratory outcomes.

This is the first prospective cohort study exploring the impact of TAI, S and FF vitamin D levels, and *VDR* expression in GCs on IVF laboratory outcomes among patients undergoing IVF/ICSI treatment. Further, we analyzed the largest cohort of patients for FF vitamin D levels and *VDR* expression. Nevertheless, the present study did have certain limitations. First, it was a single-center study; however, IVF-ET quality control and laboratory measurements are better performed in a single center rather than multiple centers. Additionally, the Center of Reproductive Medicine at Peking University Third Hospital performs >15,000 cycles of IVF-ET per year. Participants in the study were from northern and southern China, resulting in a geographically representative study population. Second, Han Chinese women were the main study participants, necessitating the enrollment of other demographic groups in the future to validate our findings. Third, data on outdoor activity conditions, seasonal factors, and daylight hours were not collected. Finally, according to the inclusion criteria, all of the patients recruited in our study were those with male or tubal factor infertility; infertile patients with an unexplained cause and/or another cause of secondary infertility were not considered within the scope of the study.

## 5 Conclusion

Vitamin D levels in the FF were correlated with those in the serum. Linear regression analysis indicated that TAI negatively affected the maturation of good-quality embryos. *VDR* expression in GCs was positively correlated with FF vitamin D levels in TAI and non-TAI patients. Considering the function of vitamin D, TAI may have a detrimental influence on IVF/ICSI laboratory outcomes.

## Data availability statement

The raw data supporting the conclusions of this article will be made available by the authors, without undue reservation.

## Ethics statement

The studies involving human participants were reviewed and approved by Peking University Third Hospital Medical Science Research Ethics committee. The patients/participants provided their written informed consent to participate in this study.

## Author contributions

YL, ZH, NH, and HC conceptualized the study, and all the authors contributed to the research discussion. YL, ZH, FM, LZ, and RL participated in patient follow-up and performed data analysis. YL wrote the initial draft of the paper and all authors contributed to the manuscript revision. All authors contributed to the article and approved the submitted version.

## References

[1] Gil Á Plaza-Diaz J Mesa MD. Vitamin d: classic and novel actions. Ann Nutr Metab (2018) 72:87–95. doi: 10.1159/000486536 29346788

[2] Nandi A Sinha N Ong E Sonmez H Poretsky L. Is there a role for vitamin d in human reproduction? Horm Mol Biol Clin Investig (2016) 25:15–28. doi: 10.1515/hmbci-2015-0051 26943610

[3] Thill M Becker S Fischer D Cordes T Hornemann A Diedrich K. Expression of prostaglandin metabolising enzymes COX-2 and 15-PGDH and VDR in human granulosa cells. Anticancer Res (2009) 29:3611–8. 19667156

[4] Yao X Wang Z El-Samahy MA Ren C Liu Z Wang F. Roles of vitamin d and its receptor in the proliferation and apoptosis of luteinised granulosa cells in the goat. Reprod Fertil Dev (2020) 32:335–48. doi: 10.1071/RD18442 31708013

[5] Antunes RA Mancebo ACA Reginatto MW Deriquehem VAS Areas P Bloise E. Lower follicular fluid vitamin d concentration is related to a higher number of large ovarian follicles. Reprod BioMed Online (2018) 36:277–84. doi: 10.1016/j.rbmo.2017.12.010 29361453

[6] Ciepiela P Dulęba AJ Kowaleczko E Chełstowski K Kurzawa R. Vitamin d as a follicular marker of human oocyte quality and a serum marker of *in vitro* fertilization outcome. J Assist Reprod Genet (2018) 35:1265–76. doi: 10.1007/s10815-018-1179-4 PMC6063829 29774457

[7] Irani M Merhi Z. Role of vitamin d in ovarian physiology and its implication in reproduction: a systematic review. Fertil Steril (2014) 102:460–8. doi: 10.1016/j.fertnstert.2014.04.046 24933120

[8] Lorini R Gastaldi R Traggiai C Perucchin PP. Hashimoto's thyroiditis. Pediatr Endocrinol Rev (2003) 1:205–11. 16444160

[9] Chao G Zhu Y Fang L. Correlation between hashimoto's thyroiditis-related thyroid hormone levels and 25-hydroxyvitamin d. Front Endocrinol (Lausanne) (2020) 11:4. doi: 10.3389/fendo.2020.00004 32117049 PMC7034299

[10] Štefanić M Tokić S. Serum 25-hydoxyvitamin d concentrations in relation to hashimoto's thyroiditis: a systematic review, meta-analysis and meta-regression of observational studies. Eur J Nutr (2020) 59:859–72. doi: 10.1007/s00394-019-01991-w 31089869

[11] Simsek Y Cakır I Yetmis M Dizdar OS Baspinar O Gokay F. Effects of vitamin d treatment on thyroid autoimmunity. J Res Med Sci (2016) 21:85. doi: 10.4103/1735-1995.192501 28163731 PMC5244647

[12] Sinclair D. Clinical and laboratory aspects of thyroid autoantibodies. Ann Clin Biochem (2006) 43:173–83. doi: 10.1258/000456306776865043 16704751

[13] van den Boogaard E Vissenberg R Land JA van Wely M van der Post JAM Goddijn M. Significance of (sub) clinical thyroid dysfunction and thyroid autoimmunity before conception and in early pregnancy: a systematic review. Hum Reprod Update (2011) 17:605–19. doi: 10.1093/humupd/dmr024 21622978

[14] Simopoulou M Sfakianoudis K Maziotis E Grigoriadis S Giannelou P Rapani A. The impact of autoantibodies on IVF treatment and outcome: A systematic review. Int J Mol Sci (2019) 20:892. doi: 10.3390/ijms20040892 30791371 PMC6412530

[15] Geva E Vardinon N Lessing JB Lerner-Geva L Azem F Yovel I. Organ-specific autoantibodies are possible markers for reproductive failure: A prospective study in an in-vitro fertilization-embryo transfer programme. Hum Reprod (1996) 11:1627–31. doi: 10.1093/oxfordjournals.humrep.a019458 8921105

[16] Hao Y Long X Kong F Chen L Chi H Zhu X. Maternal and neonatal outcomes following blastocyst biopsy for PGT in single vitrified–warmed embryo transfer cycles. Reprod BioMed Online (2021) 44:151–61. doi: 10.1016/j.rbmo.2021.07.0161472-6483 34866000

[17] Jirge PR. Poor ovarian reserve. J Hum Reprod Sci (2016) 9:963–9. doi: 10.4103/0974-1208.183514 PMC4915288 27382229

[18] Anifandis GM Dafopoulos K Messini CI Chalvatzas N Liakos N Pournaras S. Prognostic value of follicular fluid 25-OH vitamin d and glucose levels in the IVF outcome. Reprod Biol Endocrinol (2010) 8:91. doi: 10.1186/1477-7827-8-91 20667111 PMC2915999

[19] Aleyasin A Hosseini MA Mahdavi A Safdarian L Fallahi P Mohajeri MR. Predictive value of the level of vitamin d in follicular fluid on the outcome of assisted reproductive technology. Eur J Obstet Gynecol Reprod Biol (2011) 159:132–7. doi: 10.1016/j.ejogrb.2011.07.006 21835540

[20] di Clemente N Racine C Pierre A Taieb J. Anti-müllerian hormone in female reproduction. Endocr Rev (2021) 42:753–82. doi: 10.1210/endrev/bnab012 33851994

[21] Wojtusik J Johnson PA. Vitamin d regulates anti-mullerian hormone expression in granulosa cells of the hen. Biol Reprod (2012) 86:91. doi: 10.1095/biolreprod.111.094110 22174023

[22] Bednarska-Czerwińska A Olszak-Wąsik K Olejek A Czerwiński M Tukiendorf AA. Vitamin d and anti-mullerian hormone levels in infertility treatment: the change-point problem. Nutrients (2019) 11:1053. doi: 10.3390/nu11051053 31083424 PMC6567253

[23] Merhi Z Doswell A Krebs K Cipolla M. Vitamin d alters genes involved in follicular development and steroidogenesis in human cumulus granulosa cells. J Clin Endocrinol Metab (2014) 99:E1137–45. doi: 10.1210/jc.2013-4161 PMC4037738 24628555

[24] Farzadi L Khayatzadeh Bidgoli H Ghojazadeh M Bahrami Z Fattahi A Latifi Z. Correlation between follicular fluid 25-OH vitamin d and assisted reproductive outcomes. Iran J Reprod Med (2015) 13:361–6. PMC4555056 26330851

[25] Ozkan S Jindal S Greenseid K Shu J Zeitlian G Hickmon C. Replete vitamin d stores predict reproductive success following in vitro fertilization. Fertil Steril (2010) 94:1314–9. doi: 10.1016/j.fertnstert.2009.05.019 PMC2888852 19589516

[26] Huang N Chen LX Lian Y Wang HN Li R Qiao J. Impact of thyroid autoimmunity on in vitro fertilization/intracytoplasmic sperm injection outcomes and fetal weight. Front Endocrinol (2021) 12:698579. doi: 10.1016/j.fertnstert.2009.05.019 PMC8296807 34305818

[27] Huang N Liu D Lian Y Chi H Qiao J. Immunological microenvironment alterations in follicles of patients with autoimmune thyroiditis. Front Immunol (2021) 12:770852. doi: 10.3389/fimmu.2021.770852 34868029 PMC8635509

[28] Poppe K Velkeniers B Glinoer D. The role of thyroid autoimmunity in fertility and pregnancy. Nat Clin Pract Endocrinol Metab (2008) 4:394–405. doi: 10.1038/ncpendmet0846 18506157

[29] Kim D. The role of vitamin d in thyroid diseases. Int J Mol Sci (2017) 18:1949. doi: 10.3390/ijms18091949 28895880 PMC5618598

